# Serum Vitamin D Levels in Relation to Abdominal Obesity in Children and Adolescents: A Systematic Review and Dose-Response Meta-Analysis

**DOI:** 10.3389/fnut.2022.806459

**Published:** 2022-02-16

**Authors:** Zahra Hajhashemy, Keyhan Lotfi, Zahra Heidari, Parvane Saneei

**Affiliations:** ^1^Students' Scientific Research Center, Isfahan University of Medical Sciences, Isfahan, Iran; ^2^Department of Community Nutrition, School of Nutrition and Food Science, Food Security Research Center, Isfahan University of Medical Sciences, Isfahan, Iran; ^3^Department of Community Nutrition, School of Nutritional Sciences and Dietetics, Tehran University of Medical Sciences, Tehran, Iran; ^4^Department of Biostatistics and Epidemiology, School of Health, Isfahan University of Medical Sciences, Isfahan, Iran

**Keywords:** children, adolescents, serum 25-hydroxy vitamin D, abdominal obesity, meta-analysis

## Abstract

**Background:**

Findings of epidemiological studies that investigated the relationship between serum vitamin D levels and abdominal obesity were inconsistent. To evaluate the relationship between blood vitamin D levels and abdominal obesity in children and adolescents, we did a comprehensive review and dose-response meta-analysis.

**Methods:**

A comprehensive search in electronic databases including Scopus, Web of Science (ISI), MEDLINE (Pubmed), EMBASE, and Google Scholar was conducted, up to May 2021, for epidemiological studies that investigated the linkage between serum vitamin D levels (as the exposure) and abdominal obesity (as the outcome) in children and adolescents.

**Results:**

Combining 19 effect sizes from 14 cross-sectional studies that included 29,353 apparently healthy children illustrated that the highest vs. lowest level of serum vitamin D was related to a 35% reduced odds of abdominal obesity [odds ratio (OR): 0.65; 95% CI: 0.50, 0.84]. Linear dose-response analysis revealed that each 10 ng/ml increase in serum vitamin D levels was related to a 7% decrease in odds of abdominal obesity (OR: 0.93; 95% CI: 0.90, 0.95), only among investigations that used percentiles of waist circumference (>75th or 90th) to define the disorder (including 6,868 total subjects and 1,075 cases with abdominal obesity). Increasing serum vitamin D levels from 20 to 40 ng/ml was related to reduce odds of abdominal obesity in children.

**Conclusion:**

A negative relationship between blood vitamin D levels and abdominal obesity in children and adolescents was discovered in this meta-analysis of epidemiologic studies. Among investigations that used waist circumference percentiles to define the disorder, the relationship was in a dose-response manner. To affirm this relationship, more research studies are needed, particularly using a prospective design.

**Systematic Review Registration:**

https://www.crd.york.ac.uk/prospero/display_record.php?ID=CRD42021261319, PROSPERO 2021, identifier: CRD42021261319.

## Introduction

The problem of obesity and abdominal obesity affects children and adolescents around the world, both in developed and developing countries (1, 2). Considering the high prevalence of abdominal obesity in both the normal-weight and overweight children groups and its strong relation with cardiometabolic risk factors (3), measurement of waist circumference (WC) has become a routine clinical practice to prevent and manage central adiposity-related health risks (4). Several risk factors such as obesity in both parents, low educational level and smoking habits in parents, high birth weight of infants, physical inactivity (5), and nutrition status (6) are involved in the etiology of abdominal obesity in pediatrics.

Earlier investigations documented that vitamin D status of the body could be involved in the etiology of central adiposity; such that the prevalence of central adiposity could be higher in participants with vitamin D deficiency (7–9). Meanwhile, the high prevalence of vitamin D deficiency, defined as 25OHD concentrations below 20 ng/ml (or 50 nmol/l), is a major health concern due to its severe health effects (10). In Asian countries, more than half of the population suffers from vitamin D deficiency (11). In addition, the prevalence of vitamin D deficiency or insufficiency was 24 and 40% in American and European populations, respectively (12). In spite of sufficient sun exposure, vitamin D deficiency occurs more frequently in children and adolescents with sedentary lifestyles and poor dietary intake (13).

The link between blood vitamin D and abdominal obesity in children has been examined in a number of research, but the results have been inconsistent. Some studies documented an inverse significant relation between serum vitamin D and abdominal obesity in children and adolescents (7–9); however, some others could not find a significant linkage (14–16). In a recently published systematic review and meta-analysis of adult studies, we found that higher serum vitamin D levels are associated with a lower odds of abdominal obesity (17). Nevertheless, there is no comprehensive review investigating this relationship in children and adolescents. Therefore, this study summarized the link between serum vitamin D levels and abdominal obesity in children and adolescents as a result of a systematic review and meta-analysis.

## Methods and Materials

### Search Strategy

A comprehensive search of all the published papers in electronic databases including Scopus, Web of Science (ISI), MEDLINE (Pubmed), EMBASE, and Google Scholar was conducted, up to May 2021. Language and publishing year were not restricted. Details of applied MeSH and non-MeSH keywords in the systematic search are shown in Supplementary Table 1. Additional studies were investigated by a manual search of bibliographies of relevant research. In this study, we followed the Preferred Reporting Items for Systematic Reviews and Meta-Analyses (PRISMA) guidelines. CRD42021261319 was assigned to the research protocol on Prospero.

### Inclusion Criteria

We considered the following criteria to include the eligible published articles in the systematic review and meta-analysis: (1) population-based epidemiological studies with the cohort, cross-sectional or case-control design were eligible; (2) children and adolescents (<18 years) were investigated; (3) considered serum 25-hydroxy vitamin D concentration as the exposure; (4) considered abdominal obesity as the outcome of interest and (5) odds ratios (ORs), hazard ratios (HR), or relative risks (RRs) with 95% CIs were reported the relation. PICOS criteria for inclusion of studies are shown in Table 1.

**Table 1:** Population, Intervention, Comparison, Outcomes and Study (PICOS) criteria for inclusion of studies.

**Parameter**	**Criteria**
Participants	Children and adolescents (<18 years)
Intervention/Exposure	Different categories of serum vitamin D levels
Control/Comparison	Individuals in the lowest category of serum vitamin D level
Outcome	Abdominal obesity including, elevated waist circumference and waist to height ratio (WHtR) ≥0.50
Study design	Observational studies including prospective cohort, cross-sectional and case-control studies

### Exclusion Criteria

**Figure 1:**
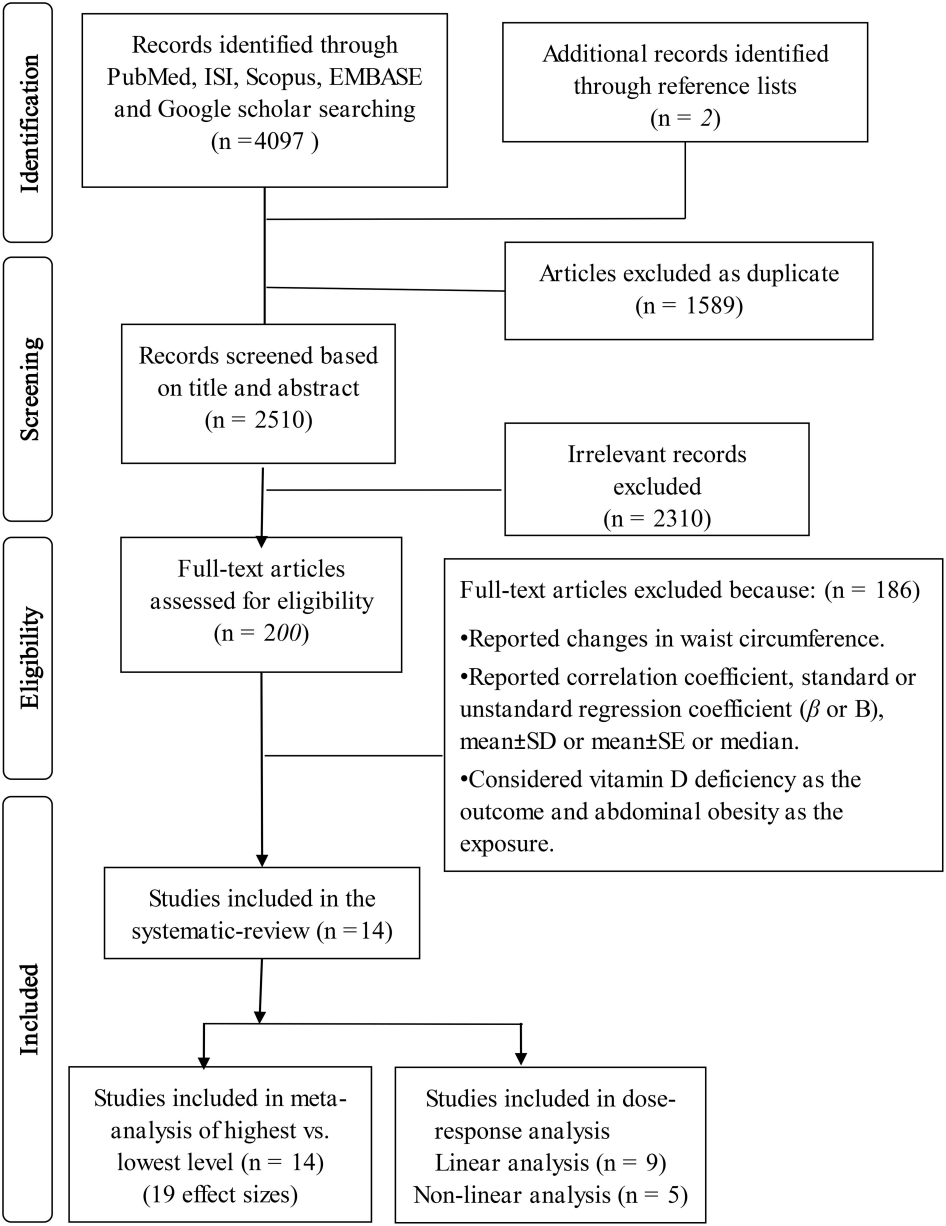
Flow diagram of the search strategy and study selection.

Supplementary Table 2 contains the details of other relevant studies that were eliminated. We excluded investigations if they: (1) reported standard or unstandard regression coefficient (β or *B*), correlation coefficient, mean ± SD or mean ± SE or median (interquartile range) for WC in various categories of serum vitamin D; (2) reported changes in WC as the outcome and did not report the risk/odds of abdominal obesity; (3) considered abdominal obesity as the exposure and vitamin D deficiency as the outcome. Moreover, we had two reports from the Korea National Health and Nutrition Examination Survey (KNHANES) 2008–2009 (18, 19) and the National Health and Nutrition Examination Survey (NHANES) 2001–2006 (9, 20). Therefore, in each pair of these investigations, only the publication with a higher sample size (9, 18) was included in the current analysis. In total, 4,097 reports were obtained from our initial systematic search. After excluding duplicate studies, two investigators (ZH and KL) separately screened the titles and abstracts of 2,510 remaining studies in the first round of screening. After that, in the second stage of screening, the complete text of 200 articles was evaluated. At the end, 14 publications were found that were eligible to be included in the current systematic review. The principal investigator (PS) supervised all the processes of screening. Figure 1 shows the flow diagram of the search strategy and study selection in further detail.

### Data Extraction

We extracted the following data from each included investigation: study design, year of publication, the last name of first author, country or study location, latitude, longitude, developing status of countries, number of subjects, health status of subjects, representativeness of the study population, unit of serum vitamin D, age range or mean age of participants, 25 (OH)D levels, sex, methods of serum vitamin D measurement, the definition of abdominal obesity, cutpoints used for defining abdominal obesity, ORs/RRs/HRs and 95% CIs for the relation of serum vitamin D with abdominal obesity, and adjustments for confounders. In addition, for the dose-response meta-analysis, the following data were collected from each eligible study: the number of participants and number of cases with abdominal obesity in each category of serum vitamin D, mean or median of serum vitamin D levels in each category, and ORs/RRs/HRs with 95% CIs for each category of circulation vitamin D for linear dose-response analysis and at least three exposure categories for nonlinear dose-response analysis. Two researchers (ZH and KL) separately extracted data. Data extraction was supervised by the principal researcher (PS).

### Quality Assessment of Studies

Using the Newcastle–Ottawa Scale (NOS), the quality of the studies that were included in the study was evaluated. Each cross-sectional study received a maximum score of 10 points: five for selection (satisfaction of sample size, description of non-respondents, representativeness of study population, ascertainment of serum vitamin D levels as the exposure), two for comparability (adjustment for main confounders including age, sex, and physical activity), and three for examining the results (utilizing a statistical test that is acceptable for the analysis and a validated evaluation of abdominal obesity as outcome). Supplementary Table 3 shows the results of the quality evaluation of the included studies. In this research, studies had a median score of 7 or more were judged as good quality, while those with a score of <7 were considered low quality. Any discrepancy was resolved by discussion.

### Statistical Analysis

The log OR and its SE were calculated using the provided ORs and their 95% CIs. Because some studies used the highest level of serum vitamin D as the reference and reported the OR for the lowest level of serum 25(OH) D, the OR and its lower and upper limits were inverted to use the lowest level of serum vitamin D as the reference and compute the OR for the highest vs. lowest levels. Regarding one included study reported linear relationship, we used the approach previously published by Danesh et al. (21) to convert the linear relationship between blood vitamin D and abdominal obesity (22) to T3 vs. T1 of serum vitamin D. For the mentioned study that reported odds of abdominal obesity for one unit increase in serum vitamin D levels, we assumed a normal distribution for log OR and multiplied log OR to the factors of 2.18 and SD to have OR for the third vs. first category of serum vitamin D. The overall estimate was calculated using a random-effects model that took into account between-study variation. To measure between-study heterogeneity, the Cochran's *Q*-test and *I*^2^ were employed. When there was considerable between-study heterogeneity, subgroup analyses were used to investigate the probable source of heterogeneity based on different confounders including, quality of studies, levels of vitamin D used for comparison, sex, cutoff-points used for defining abdominal obesity, study location (Asian vs. non-Asian countries) and (developed vs. developing countries), methods of vitamin D measurement and adjustment for age and physical activity. To determine heterogeneity between subgroups, a fixed-effect model was utilized. For continuous variables (including quality of studies, age, latitude, and longitude), meta-regression was also conducted. Sensitivity analysis was used to see how much inferences were based on a single research. Visual inspection of Begg's funnel plots and statistical assessment of its funnel plot asymmetry by Begg's test and Egger's test were used to assess publication bias.

Greenland and Longnecker (23) and Orsini et al. (24) techniques for dose-response analysis were also used. In this method, we required the total number of participants, number of participants with abdominal obesity and mean or median of serum vitamin D for each category. Using the natural logs of the ORs and 95% CIs across categories of serum vitamin D, study-specific slopes (linear trends) and nonlinear trends 95% CIs were computed. For nonlinear trends, we required at least three quantitative categories of exposure and used restricted cubic splines (three knots at fixed percentiles of 10, 50, and 90% of the distribution). The matching OR of each group was given to the mean (or median) level of blood vitamin D in that category. The midpoint of each category was calculated by taking the average of the lower and upper bounds in studies that reported serum 25(OH) D levels as ranges. In the case of open-ended highest category, we assumed the length of open-ended interval as the same as that of the adjacent interval. The lower threshold for 25(OH) D was set to zero when the lowest category was left open-ended. For statistical analyses, Software for Statistics and Data Science (STATA) version 14.0 (STATA Corporation, College Station, Texas, USA) was used. For all the tests, including Cochran's Q, *P*-values of 0.05 were deemed statistically significant.

## Results

### Study Characteristics

Overall, 14 eligible investigations were included in the systematic review and meta-analysis; details of these investigations are shown in Table 2. These studies were published between 2011 and 2020. All the included publications had cross-sectional designs and investigated 29,353 children and adolescents. Four of these cross-sectional studies were carried out in South Korea (7, 15, 18, 22), 2 in China (16, 25), Saudi Arabia (26, 27) and the remaining in Iran (29), USA (9), Portugal (14), Chile (8), Spain (28), and Italy (30). Nine of them were conducted in Asian countries (7, 15, 16, 18, 22, 25–27, 29) and five others were carried out in non-Asian regions (8, 9, 14, 28, 30). Nine investigations (7, 9, 12, 15, 16, 18, 22, 27, 28, 30) have considered WC ≥90th percentile as abdominal obesity; two others (8, 14) considered WC ≥ 75th percentile and the three remaining studies (25, 26, 29) considered waist to height ratio (WHtR) ≥0.50 or 0.56 to define abdominal obesity in children and adolescents. Eligible studies have used different methods for serum vitamin D measurement including, chemiluminescent immunoassay (CLIA) (*n* = 6) (7, 14, 25, 26, 28, 29), radioimmunoassay (RIA) (*n* = 3) (9, 18, 22), immunoassay (IA) (*n* = 2) (8, 27), electrochemiluminescence immunoassay (ECLIA) (*n* = 1) (30), enzyme immunoassay (EIA) (*n* = 1) (16); although one investigation did not report the using method (15). Among the included studies, 11 studies made the adjustment for age and five studies for physical activity. Almost all investigations have randomly selected their subjects and their samples were representative of the whole children population, except one study (30) that did not use the random-sampling method. Five eligible publications have separately reported the relation in boys and girls; whereas others reported their estimates for both genders together. Nine of these cross-sectional investigations were high quality, while five others were low quality.

**Table 2:** Main characteristics of included studies examined the relation between serum vitamin D levels and abdominal obesity in children and adolescents.

**References**	**Study design/name study**	**Country latitude, **°***N***	**Age range/ Mean age**	**Sex**	**No. Participants**	**25 (OH)D Levels, nmol/L**	**OR, (95% CI)**	**Method (Exposure)**	**Definition (Outcome)**	**Subject**	**Adjustment**
Tang et al. (15)	Cross-sectional (survey of a school-based interventional project)	South China	7–18/10	Both	2,112	<16.19 ng/ml 16.20–19.74 19.75–22.99 ≥23	1.26 (0.87–1.83) 1.08 (0.74–1.58) 0.98 (0.67–1.44) 1(Ref)	EIA	WC > 90th percentile for age and gender	Chinese children and adolescents	1–7
Xiao et al. (25)	Cross-sectional	China	6–18	Boys	3,057	<30 nmol/l 30–50 ≥50	0.78 (0.49–1.23) 1.11 (0.76–1.64) 1(Ref)	CLIA	WHtR ≥ 0.50	children and adolescents	1, 3, 8–15
				Girls	3,034	<30 nmol/l 30–50 ≥50	1.22 (0.80–1.86) 1.15 (0.78–1.69) 1(Ref)				
Fu et al. (9)	Cross-sectional (NHANES 2001–2006)	USA	6–18/12.8	Both	6,260	<30 nmol/l 30–50 >50	3·38 (2·60–4·39) 2·28 (1·93–2·68) 1(Ref)	RIA	WC ≥ 90th percentile	US children	2, 1, 11, 16–20
Kim et al. (15)	Cross-sectional (KNHANES 2010–2014)	Korea	12–18/15.14 ± 0.3	Both	2,314	≤ 20 ng/ml >20	1.08 (0.58–2.02) 1(Ref)	NR	WC ≥ 90th percentile (if ≥16 years, boys ≥90 cm, girls ≥ 85 cm)	Korean adolescents	1–3, 9, 20–24
Cabral et al. (14)	Cross-sectional (EPITeen 2003–2004)	Portugal	13	Both	514	<13.0 ng/ml 13.0–16.0 17.0–20.0 >20.0	1.26 (0.48–3.29) 1.03 (0.36–2.89) 1.64 (0.58–4.50) 1(Ref)	CLIA	WC ≥ 75th percentile for age and gender	Adolecsents	2, 3, 8, 13, 25
Cediel et al. (8)	Cross-sectional (GOCS)	Santiago, Chile	8.0 ± 1.3 6.3 ± 0.6	Boys Girls	203 232	<30 ng/ml ≥30 <30 ng/ml ≥30	2.2 (1.2–4.0) 1(Ref) 2.4 (1.4–4.3) 1(Ref)	IA	WC ≥ 75th percentile (NHANES III percentiles Mexican–Children: cut-off ≥75th percentile in girls at 6 years: 60.4 cm; and boys at 8 years: 66.2 cm)	Prepubertal chilean children	1, 8
Al-Daghri et al. (26)	Cross-sectional	Riyadh, Saudi Arabia	12–17/14.3	Boys	1,906	<25 nmol/l 25–50 ≥50	1.30 (0.49–3.43) 1.55 (0.73–3.31) 1(Ref)	CLIA	WHtR > 0.56	Apparently healthy Saudi school students	–
				Girls	2,277	<25 nmol/l 25–50 ≥50	1.45 (0.66–3.19) 1.19 (0.54–2.65) 1(Ref)				
Al-Daghri et al. (27)	Cross-sectional	Riyadh, Saudi Arabia	13–17/15.1	Boys	1,187	<25 nmol/l 25–49.9 >50	(0.32–3.10) 2.75 (1.1–7.1) 1(Ref)	IA	WC > 90th percentile (>92 cm for boys and >86 cm for Girls)	Saudi adolescents	1, 13, 26–30
				Girls	1,038	<25 nmol/l 25–49.9 >50	1.31 (0.38–4.45) 1.27 (0.36–4.42) 1(Ref)				
De Piero Belmonte et al. (28)	Cross-sectional	Spain	8–13/10.7 ± 1.0	Both	314	4.0–19.4 ng/mL 19.5–25.3 25.4–55.5	1(Ref) 1.14 (0.63–2.05) 0.77 (0.56–1.07)	CLIA	WC ≥ 90th percentile	Spanish school-children	1, 2
Jari et al. (29)	Cross-sectional (CASPIAN-III study)	27 provinces in Iran	10–18	Boys	568	<10 ng/ml 10–30 ≥30	1.07 (0.55, 2.05) (0.56–2.14) 1(Ref)	CLIA	WHtR >0.5	Students	1, 9
				Girls	527	<10 ng/ml 10–30 ≥30	0.69 (0.35, 1.35) 0.91 (0.48, 1.74) 1(Ref)				
Nam et al. (18)	Cross-sectional (KNHANES 2008–2009)	South Korea	12–18/	Both	1,504	≤ 50 nmol/l >50	2.05 (1.20-3.49) 1(Ref)	RIA	WC ≥ 90th percentile for age and gender	Adolecsents	1–3, 11, 31
Lee et al. (22)	Cross-sectional (Ewha Birth and Growth Cohort study 2001–2006)	Korea	7–9/7.89	Both	205	Per 1 ng/mL increase of 25(OH)D	0.87 (0.75–1.01)	RIA	WC ≥ 90th percentile	Preadolescent children	1, 2, 25, 32, 33
Lee et al. (7)	Cross-sectional (KMOSES) 2006–2010)	South Korea	9	Both	1,649	<15.5 ng/ml 15.5–18.3 18.4–21.6 >21.6	2.96 (1.75–5.00) 2.32 (1.36–3.95) 2.08 (1.20–3.60) 1(Ref)	CLIA	WC > 90th percentile for age and gender	Children	13
Pacifico et al. (30)	Cross-sectional	Rome, Italy	11.2	Both	452	<17.0 ng/ml 17.0–27.0 >27	1.98 (0.83–4.73) 1.20 (0.75–1.91) 1(Ref)	ECLIA	WC ≥ 90th percentile for age and gender	Caucasian children and adolescents	1, 2, 13, 34

*Ref, reference; WC, waist circumference; IA, immunoassay; CLIA, chemiluminescent immunoassay; EIA, enzyme-immunosorbent assay; RIA, radioimmunoassay; ECLIA, electrochemiluminescence immunoassay; NHANES, national health and nutrition examination surveys; EPITeen, epidemiological health investigation of teenagers; KNHANES, Korea national health and nutrition examination survey; GOCS, growth and obesity Chilean cohort study; CASPIAN, childhood and adolescence surveillance and prevention of adult noncommunicable disease; KMOSES, Korean metabolic disorders and obesity study in elementary school Childre; WHtR, waist to height ratio. Adjustments: 1, Age; 2, sex; 3, physical activity; 3, sun exposure time; 4, screen time; 5, intake of fruit; 6, intake vegetables; 7, intake of meat products; 8, season of blood collection; 9, geographical location; 10, smoking; 11, drinking; 12, dietary vitamin D intake; 13, Body mass index (BMI); 14, fat mass percentage (FMP); 15, muscle mass index (MMI); 16, cotinine concentration; 17, race; 18, ethnicity; 19,poverty; 20, income ratio; 21, self-perceived health status; 22, self-perceived stress status; 23, family history of chronic disease; 24, sleep; 25, parental education; 26, glucose; 27, total cholesterol; 28, triglycerides; 29, High-density lipoproteins cholesterol (HDL-c); 30, Low- density lipoproteins cholesterol (LDL-c); 31, use of multivitamin or mineral supplement; 32, birth order; 33,fruit/fruit juice intake; 34,Tanner stage*.

### Finding From Meta-Analysis of the Highest vs. Lowest Level of Serum Vitamin D in Relation to Abdominal Obesity in Children

**Figure 2:**
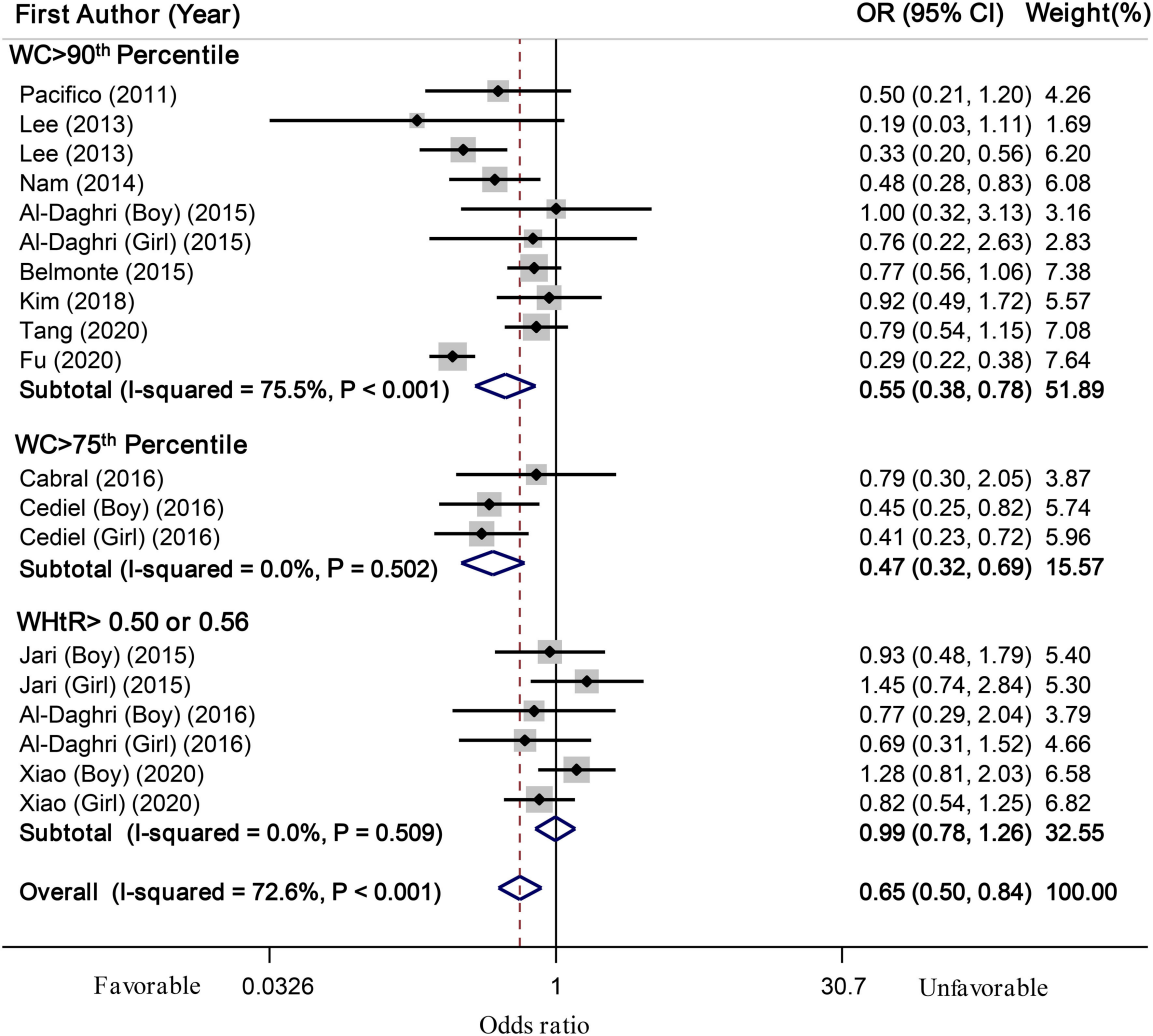
Forest plots of the relationship between serum vitamin D levels and abdominal obesity in children and adolescents, stratified by abdominal obesity definition.

In total, 19 effect sizes from 14 eligible studies (including 29,353 children and adolescents) were included in this meta-analysis. Results showed that the highest serum vitamin D level was significantly related to a 35% decreased odds of abdominal obesity in children and adolescents, compared to the lowest level (OR: 0.65; 95% CI: 0.50, 0.84; Figure 2). However, there was significant heterogeneity between studies (*I*^2^ = 72.6%, *P* < 0.001); therefore, we applied subgroup analysis based on abdominal obesity definition to explore the probable source of heterogeneity. Among two subgroups that considered WC > 90th percentile and WC > 75th percentile as abdominal obesity, serum vitamin D level was significantly related to lower odds of abdominal obesity [OR for WC > 90th percentile: 0.55, (95% CI: 0.38, 0.78) and OR for WC > 75th percentile: 0.47, (95% CI: 0.32, 0.69)]; however, in subgroup of studies that used WHtR > 0.50 or 0.56 to define abdominal obesity, no significant relation was found (OR: 0.99; 95% CI: 0.78, 1.26). Although, between-study heterogeneity was removed in subgroups of “WC> 75th percentile” (*I*^2^ = 0.0%, *P* = 0.50) and WHtR > 0.50 or 0.56 (*I*^2^ = 0.0%, *P* = 0.51), it was still significant in subgroup of “WC > 90th percentile” (*I*^2^ = 75.5%, *P* < 0.001; Figure 2). Thus, subgroup analyses were conducted based on other confounders including sex, study location (Asian vs. non-Asian countries), development status of countries, quality score, methods of vitamin D measurement, vitamin D categories, and adjustment for physical activity and age. Results of these subgroup analyses are shown in Table 3. Almost all the included studies have used random-sampling models and their studies samples were representative of the whole children population, except one study that investigated a non-representative sample (30). After excluding this study with a non-representative population from the analysis, we found that high vs. low level of serum vitamin D was related to 34% lower odds of abdominal obesity in the general children population (OR: 0.66; 95% CI: 0.50, 0.86; *I*^2^ = 74.01%, *P* < 0.001). Moreover, meta-regression was conducted to examine the effect of continuous confounders on the overall estimate. Although the mean age of participants (β = 0.101, *P* = 0.04, $I_{\text{residual}}^{2}$ = 74.04%) had an effect on overall estimate, none of other covariates [including latitude (β = −0.032, *P* = 0.17, $I_{\text{residual}}^{2}$ = 68.91%), longitude (β = −0.002, *P* = 0.40, $I_{\text{residual}}^{2}=$ 72.86%), and quality score of studies (β = 0.118, *P* = 0.37, $I_{\text{residual}}^{2}$ =73.73%)] had a significant effect on overall OR. Based on sensitivity analysis no particular study significantly had a significant impact on the overall estimate. There was no evidence of significant publication bias based on visual inspection of Begg's funnel plot and statistical tests of Begg's (*P* = 0.60) and Egger's (*P* = 0.32).

**Table 3:** Results of subgroup analyses of serum vitamin D levels in relation to abdominal obesity in children and adolescent.

	**Effect sizes (*n*)**	***P*** **within^a^**	***I*** **^2^ (%)**	***P*** **between^b^**	**OR (95% CI)**
Overall	19	<0.001	72.6		0.65 (0.50, 0.84)
Gender				0.001	
Both	9	<0.001	77.7		0.53 (0.37, 0.77)
Boy	5	0.11	46.5		0.84 (0.55, 1.30)
Girl	5	0.08	51.7		0.75 (0.49, 1.15)
Adjustment for physical activity				<0.001	
Yes	6	0.18	33.0		0.82 (0.64, 1.07)
No	13	<0.001	71.8		0.57 (0.41, 0.80)
Adjustment for age				0.29	
Yes	15	<0.001	76.6		0.67 (0.50, 0.90)
No	4	0.19	35.8		0.54 (0.33, 0.88)
Asian vs. non-Asian countries				<0.001	
Asian	13	0.01	50.9		0.76 (0.59, 0.99)
Non-Asian	6	<0.001	77.3		0.49 (0.32, 0.75)
Developed vs. developing countries				<0.001	
Developed	8	<0.001	76.0		0.50 (0.33,0.74)
Developing	11	0.08	39.1		0.80 (0.62, 1.01)
Quality score^c^				0.93	
Low quality (Scores ≤ 7)	6	0.09	47.1		0.55 (0.37, 0.82)
High quality (Scores > 7)	13	<0.001	78.7		0.70 (0.50, 0.98)
Methods of vitamin D measurement				<0.001	
IA and EIA	5	0.23	27.6		0.60 (0.43, 0.85)
CLIA and ECLIA	10	0.02	53.7		0.78 (0.59, 1.03)
RIA	3	0.22	32.7		0.34 (0.23, 0.49)
NR	1	–	–		0.92 (0.49, 1.72)
Vitamin D categories				0.48	
Q_4_ vs. Q_1_	3	0.02	72.8		0.58 (0.31, 1.09)
T_3_ vs. T_1_	3	0.22	33.5		0.61 (0.35, 1.05)
Sufficiency vs. deficiency	13	<0.001	77.7		0.69 (0.49, 0.98)

*CLIA, chemiluminescent immunoassay; RIA, radioimmunoassay; IA, immunoassay; ECLIA, electrochemiluminescence immunoassay; EIA, enzyme immunoassay*.

a *P for heterogeneity, within subgroup*.

b *P for heterogeneity, between subgroups*.

c *Quality Scores were according to Newcastle-Ottawa Scale*.

### Findings From Dose-Response Analysis of Serum Vitamin D in Relation to Abdominal Obesity in Children

**Figure 3:**
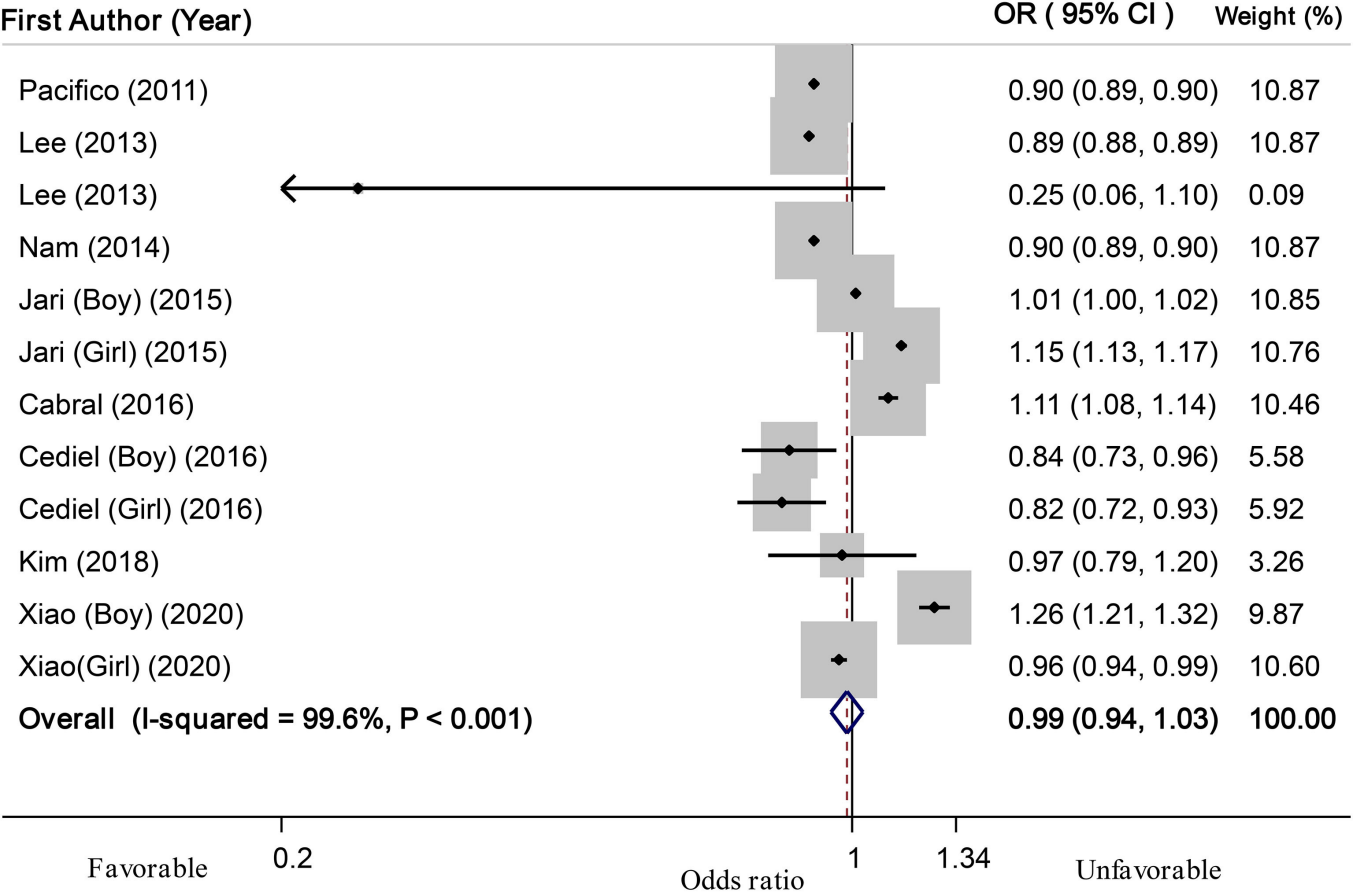
Linear dose-response meta-analysis of the relationship between each 10 ng/ml (25 nmol/L) increment in serum 25(OH) D levels and abdominal obesity in children and adolescents.

**Figure 4:**
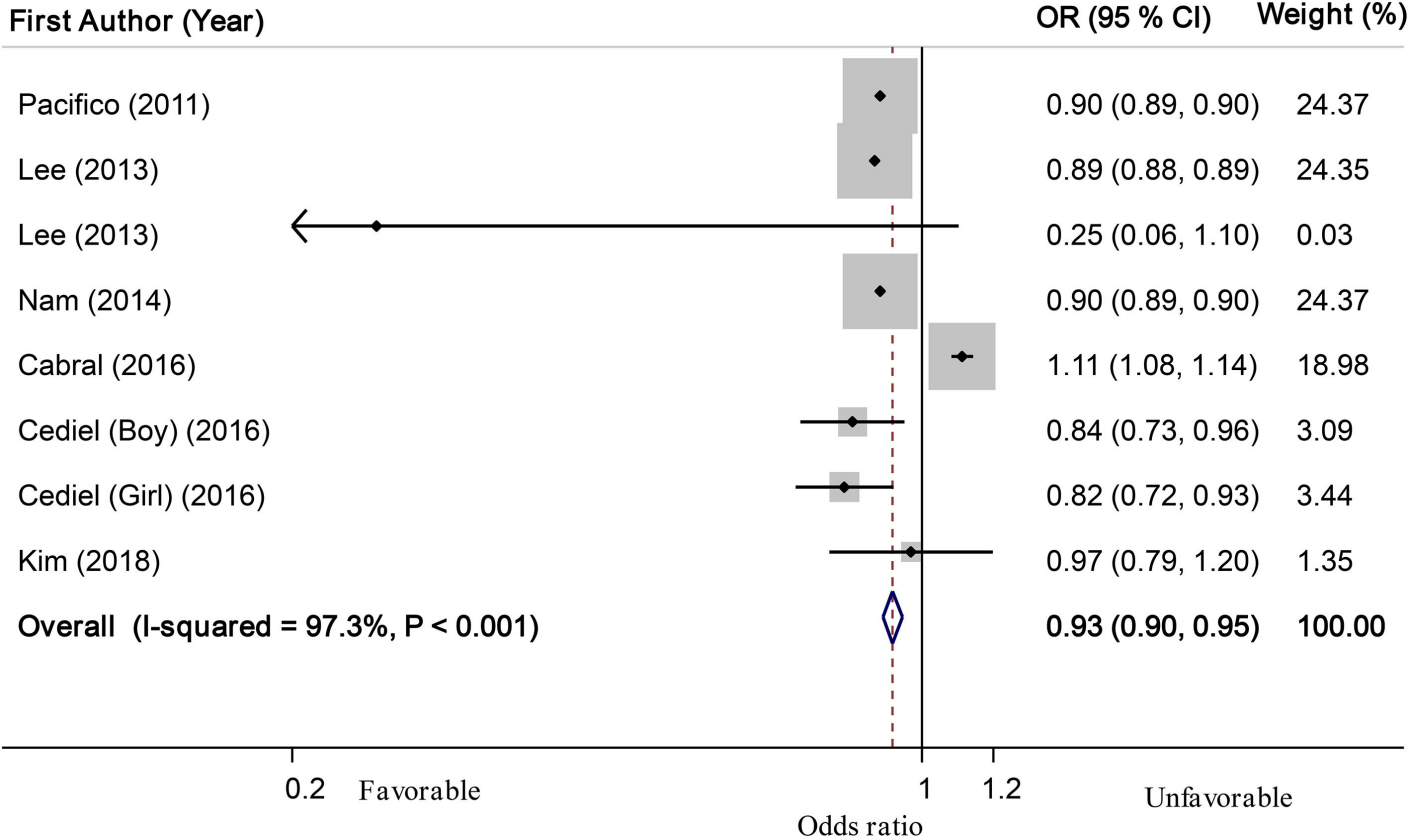
Linear dose-response meta-analysis of the relationship between each 10 ng/ml (25 nmol/L) increment in serum 25(OH) D levels and abdominal obesity in children and adolescents among studies that used waist circumference percentiles to define abdominal obesity.

**Figure 5:**
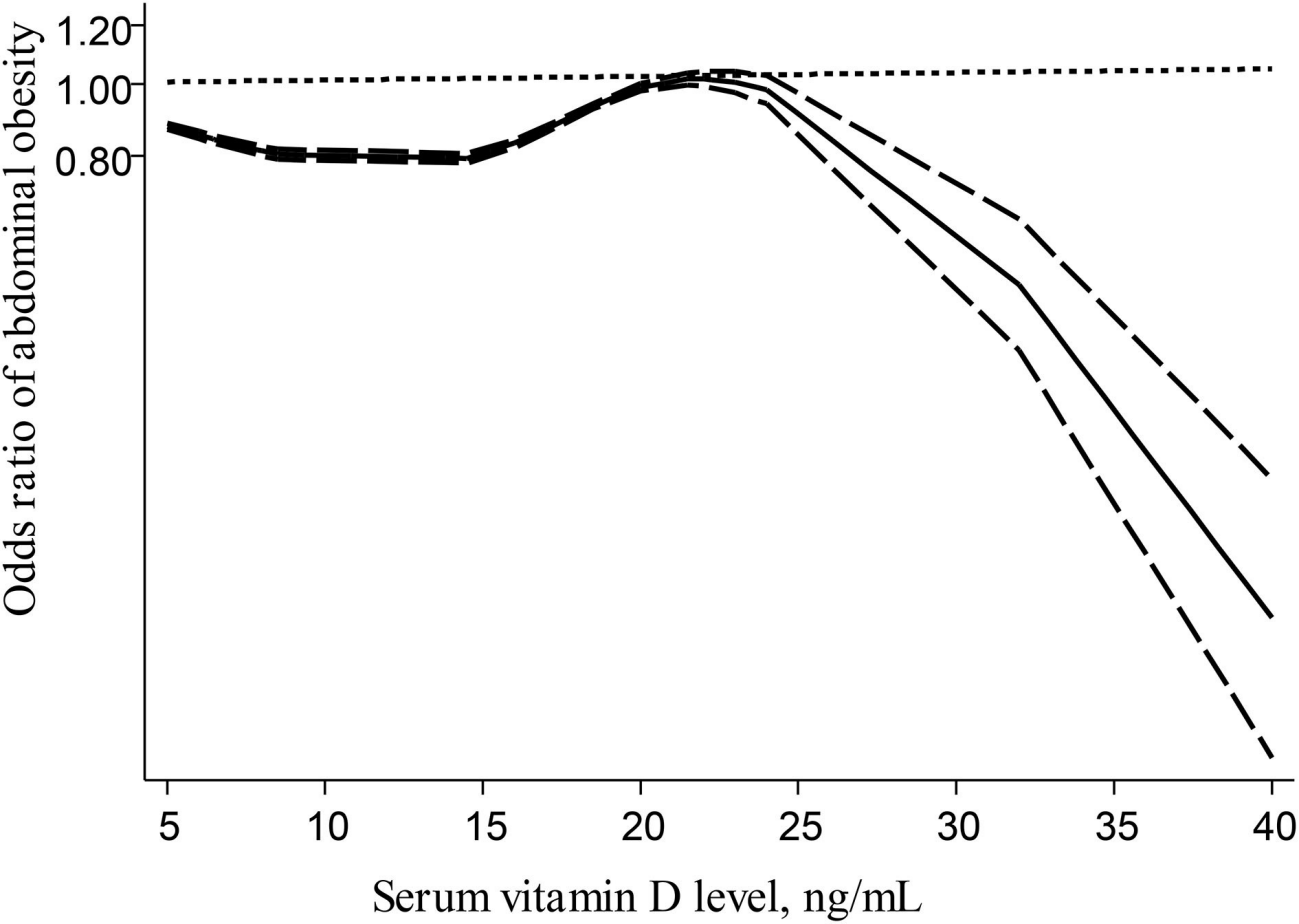
Nonlinear dose–response relationship between serum vitamin D levels and abdominal obesity in children and adolescents; - - -, Linear model; ____, spline model.

Based on linear dose-response analysis on nine eligible studies (7, 8, 14, 15, 18, 22, 25, 29, 30) (including 14,045 children or adolescents and 2,181 cases with abdominal obesity), each 10 ng/ml (or 25 nmol/l) increase in serum vitamin D levels was associated with a 1% non-significant decline in odds of abdominal obesity in children and adolescents (OR: 0.99; 95% CI: 0.94, 1.03; Figure 3). When we excluded two studies that used WHtR values to define abdominal obesity (25, 29) from linear dose-response analysis, we found that among seven investigations (7, 8, 14, 15, 18, 22, 30) (with 6,868 total participants and 1,075 cases with abdominal obesity), which used percentiles of WC for defining abdominal obesity, each 10 ng/ml increase in serum vitamin D levels could be related to a 7% decreased odds of abdominal obesity (OR: 0.93; 95% CI: 0.90, 0.95; Figure 4). Furthermore, nonlinear dose-response analysis on five eligible investigations (7, 14, 25, 29, 30) (including 9,801 total participants and 1,692 cases with abdominal obesity) was significant (*P*_nonlinearity_ < 0.001; Figure 5). Increased level of serum 25-hydroxy vitamin D from 20 to 40 ng/ml was associated with a steeper reduction in odds of abdominal obesity in pediatrics.

## Discussion

The current meta-analysis revealed that the highest level of serum vitamin D was related to lower odds of abdominal obesity in children and adolescents, in comparison with the lowest level. Subgroup analysis based on different covariates revealed that this inverse relationship was significant in most subgroups. Based on the linear dose-response analysis, each 10 ng/ml (or 25 nmol/l) increment in blood vitamin D, was related to a 7% significant decrease in odds of abdominal obesity, only among investigations that used percentiles of WC (>75th or 90th) to define the disorder. Furthermore, as blood vitamin D levels elevated from 20 to 40 ng/mL, there was a steeper drop in the odds of abdominal obesity.

Obesity and abdominal obesity in children and adolescents have grown at an alarming rate in recent decades, resulting in a variety of health issues (1, 2). Further attentions in both clinical practice and epidemiological studies are required to avoid early harmful consequences of childhood abdominal obesity (3, 31, 32). Abdominal obesity was more common in individuals with lower blood vitamin D levels in this study; thus, serum vitamin D status in children and adolescents should be managed in order to reduce the risk of abdominal obesity and its associated morbidity and mortality.

According to our findings, there was an inverse relationship between blood vitamin D levels and abdominal obesity among studies that used WC percentiles to define abdominal obesity. But, this relationship was not significant among investigations that applied WHtR values for defining abdominal obesity. Some previous investigations suggested that among growing children and adolescents, WHtR could be more useful for classifying abdominal obesity than waist circumference alone. However, both numerator and denominator of WHtR could be influenced by serum vitamin D levels (33). In addition, genetic might affect the growth of children and their final height (34). On the other hand, WC percentiles have been developed based on the national data of each country. So, using WC percentiles in routine clinical practice might better identify those with abdominal obesity and cardio-metabolic risks than WHtR values.

Some systematic reviews and meta-analyses have examined the relationship between blood vitamin D levels and non-communicable disease (NCDs) and their findings were in line with ours (17, 35–37). Based on our previous analysis, serum vitamin D was found to be inversely linked to odds of abdominal obesity in a dose-response fashion, in adults (17). In another investigation, the highest value of serum vitamin D was significantly associated with a 50% lower risk of metabolic syndrome (MetS) in children. Furthermore, for every 10 ng/mL increase in serum vitamin D, the risk of MetS in children was linearly lowered by 12% (35). Moreover, a previous meta-analysis showed a weak significant inverse correlation between serum vitamin D level and serum triglycerides (TG) levels in the pediatric age group; but no significant correlation was observed in case of low-density lipoprotein cholesterol (LDL-c), high-density lipoprotein (HDL-c) concentrations, or total cholesterol (TC) (36). A systematic review has also suggested an inverse relationship between serum vitamin D levels and systolic blood pressure (SBP) in children; but the authors could not quantify the relation of vitamin D with blood pressure or other cardio-metabolic risk factors through meta-analysis, due to the small number of eligible studies (37). Nevertheless, in the current analysis, serum vitamin D was inversely associated with odds of central adiposity in childhood abdominal, in a dose-response fashion model.

This study suggested that waist circumference might be a more useful and accurate tool to correctly measure abdominal obesity and its relation with health-disease status in children. A previous cross-sectional study has also measured trunk fat via dual-energy X-ray absorptiometry in 278 girls and 302 boys to assess the validity of waist circumference, waist-to-hip ratio (WHR), and the conicity index, which evaluates waist circumference in relation to height and weight. In line with our finding, the mentioned study showed that waist circumference was the best index to define abdominal obesity (38). Another cross-sectional study conducted on 14,500 children and adolescents demonstrated that waist circumference might be a good indicator for screening abdominal fat storage (39). Some other previous investigations have also mentioned the disadvantage of using other indices such as WHR and WHtR to define abdominal obesity (40, 41). As the reduction in body weight could proportionately decrease both the waist and hip circumference, WHR might not reflect the change in visceral fat (40). WHtR may additionally not be a good indicator, because height is inversely related to all-cause and other specific mortality and morbidity, independent of fat distribution (41).

Although previous investigations could not exactly clarify the underlying mechanisms of serum vitamin D-abdominal obesity relation in children, some pathways were proposed to explain this relation. *In vitro* studies revealed that vitamin D is essential for fat distribution through controlling adipogenesis (42) and lipolysis (43). In addition, excessive screen time (television, computers, and tablets) and consequently, low outdoor physical activity and sun exposure are involved in the etiology of both abdominal obesity and vitamin deficiency in children and adolescents (44, 45). Furthermore, vitamin D is a fat-soluble vitamin, thus having more body fat would enhance the storage of vitamin D in adipose tissue (46). Moreover, vitamin D has a key role in energy metabolism by modifying β-oxidation and uncoupling-protein expression (47). Based on previous evidence, expression of adipocyte uncoupling protein 2 (UCP-2), a factor stimulate lipogenesis and inhibit lipolysis, could be suppressed by 1, 25 (OH)_2_D_3_ (48, 49). Finally, previous research has shown that obese adolescents do not respond to vitamin D treatment in the same way that non-obese adolescents do (50, 51). As a result, children with obesity should take higher amounts of vitamin D supplements. Nonetheless, there is no universally accepted dosage for the therapy of vitamin D insufficiency in children (50, 52).

The current meta-analysis has several strengths. To our knowledge, there was no previous study that investigated the relationship between circulating 25 (OH) D levels and central adiposity in children and adolescents and this subject is novel. The study comprised a large pediatric population, and subgroup analyses were performed depending on various covariates. Almost all of the research included in this review employed random sampling methods, and the results could be applied to the whole population of children. Furthermore, our dose-response analysis showed that the inverse linear relationship between blood vitamin D and abdominal obesity in pediatrics was dependent on the definition of the disorder. In addition, the effect of potential confounders including physical activity, sex, and age were controlled in most included studies. However, some weaknesses should be considered. We were unable to compute distinct ORs for boys and girls because only one-third of eligible studies (*n* = 5) provided separate data on the relationship between circulating 25-hydroxy vitamin D and abdominal obesity in boys and girls. Some of the investigations included in this study did not take into account the effects of body mass index (BMI), and hours of sun exposure or season of blood collection in their analysis; several other studies did not consider the use of multivitamin or mineral supplements, and dietary intake of vitamin D. Additionally, use of sunscreen or treatment with certain drugs that might influence the metabolism of vitamin D could be potential confounders that none of the included studies made adjustment for them. The indicated constraints resulted in between-study heterogeneity which was not fully removed even after subgroup analysis and meta-regression. The included studies were all cross-sectional and lack of prospective data, making it impossible to draw a causal link. As the causality of this relation is not determined, it might also be possible that children and adolescents with abdominal obesity might have low serum vitamin D. To determine causation and directness of the association, further prospective studies are needed.

In conclusion, a negative relationship between blood vitamin D levels and the risk of abdominal obesity in children and adolescents was discovered in this meta-analysis of epidemiologic studies. Investigations with representative childhood populations yielded the same result. The relationship was dose-response in studies that utilized waist circumference percentiles as a criterion for defining the disorder. To affirm this relationship, more research studies are needed, particularly using prospective design.

## Data Availability Statement

The original contributions presented in the study are included in the article/Supplementary Material, further inquiries can be directed to the corresponding author/s.

## Author Contributions

ZHa, KL, ZHe, and PS contributed to the conception, design, statistical analyses, data interpretation, and manuscript drafting. PS supervised the study. All authors approved the final manuscript for submission.

## Funding

The financial support for conception, design, data analysis, and manuscript drafting comes from Isfahan University of Medical Sciences, Isfahan, Iran (No. 1400158). Isfahan University of Medical Sciences had no role in the design or conduct of the study, collection, analysis and interpretation of the data, and preparation, review, and approval of the manuscript.

## Conflict of Interest

The authors declare that the research was conducted in the absence of any commercial or financial relationships that could be construed as a potential conflict of interest.

## Publisher's Note

All claims expressed in this article are solely those of the authors and do not necessarily represent those of their affiliated organizations, or those of the publisher, the editors and the reviewers. Any product that may be evaluated in this article, or claim that may be made by its manufacturer, is not guaranteed or endorsed by the publisher.
