# Supplementary material for: Serum Vitamin D Levels in Relation to Abdominal Obesity in Children and Adolescents: A Systematic Review and Dose-Response Meta-Analysis
Source: Front Nutr. 2022 Feb 16;9:806459. doi: 10.3389/fnut.2022.806459 (PMC8888413; doi:10.3389/fnut.2022.806459)
Supplement: Supplementary file 1 [file Table_1.PDF]

Supplementary Material

Serum vitamin D levels in relation to abdominal obesity in children and adolescents: A systematic review and dose-response meta-analysis

Hajhashemy et al.

Supplemental Table 1. *MeSH and non-MeSH terms that were used in the systematic search*

Database	*Syntax*	*Results*
PubMed	*("vitamin D"[Title/Abstract] OR "25 hydroxy*"[Title/Abstract] OR cholecalciferol*[Title/Abstract] OR Hydroxycholecalciferol*[Title/Abstract] OR ergocalciferol*[Title/Abstract] OR dihydrotachysterol*[Title/Abstract] OR "25-Hydroxyvitamin D 2"[MeSH Terms] OR "vitamin D"[MeSH Terms] OR Cholecalciferol[MeSH Terms] OR hydroxycholecalciferols[Mesh Terms] OR Ergocalciferols[Mesh Terms] OR Dihydrotachysterol[Mesh Terms]) AND ("obes*"[Title/Abstract] OR "antropometric"[Title/Abstract] OR "central fat"[Title/Abstract] OR "Metabolic Syndrome"[Title/Abstract] OR "abdominal adiposit*"[Title/Abstract] OR "Overweight"[Title/Abstract] OR "central adiposity"[Title/Abstract] OR "obesity, abdominal"[MeSH Terms] OR "Waist Circumference"[MeSH Terms] OR "Metabolic Syndrome"[MeSH Terms] OR Overweight[MeSH Terms]) AND (child*[Title/Abstract] OR "infant"[MeSH Terms] OR "child"[MeSH Terms] OR "adolescent"[MeSH Terms])*	*1093*
Web Of Sciences	*TI=("vitamin D" OR "25 hydroxy*" OR "25-Hydroxyvitamin D 2" OR cholecalciferol* OR Hydroxycholecalciferol* OR ergocalciferol* OR dihydrotachysterol*) AND TS=(obes* OR antropometric OR "central fat" OR "Metabolic Syndrome" OR "abdominal adiposit*" OR Overweight OR "central adiposity") AND TS=(infant OR child* OR adolescent*)*	*726*
Scopus	*(TITLE ("vitamin D" OR "25 hydroxy*" OR "25-Hydroxyvitamin D 2" OR cholecalciferol* OR hydroxycholecalciferol* OR ergocalciferol* OR dihydrotachysterol*) AND TITLE-ABS-KEY (obes* OR antropometric OR "central fat" OR "Metabolic Syndrome" OR "abdominal adiposit*" OR overweight OR "central adiposity") AND TITLE-ABS-KEY (infant OR child* OR adolescent*))*	*794*

Embase	*(obes*:ti,ab,kw OR antropometric:ti,ab,kw OR 'central fat':ti,ab,kw OR 'metabolic syndrome':ti,ab,kw OR 'abdominal adiposit*':ti,ab,kw OR overweight:ti,ab,kw OR 'central adiposity':ti,ab,kw OR 'abdominal obesity'/exp/mj) AND (infant:ti,ab,kw OR child*:ti,ab,kw OR adolescent*:ti,ab,kw) AND ('vitamin d':ti,ab,kw OR '25 hydroxy*':ti,ab,kw OR '25-hydroxyvitamin d 2':ti,ab,kw OR cholecalciferol*:ti,ab,kw OR hydroxycholecalciferol*:ti,ab,kw OR ergocalciferol*:ti,ab,kw OR dihydrotachysterol*:ti,ab,kw OR 'vitamin d'/exp/mj OR '25 hydroxyvitamin d'/exp/mj)*	*1484*

Supplemental Table 2. *Details of more relevant studies that were excluded.*

Reference number	***First Author/ Year***	***Title of paper***	***Reason of exclusion from current systematic review and meta-analysis***
(1)	*Al-Sadat, 2016*	*Vitamin D deficiency in Malaysian adolescents aged 13 years: findings from the Malaysian Health and Adolescents Longitudinal Research Team study (MyHeARTs)*	*Considered vitamin D deficiency as the outcome and waist circumference as the exposure*
(2)	*Rodrı´guez-Rodrı´guez, 2010*	*Associations between abdominal fat and body mass index on vitamin D status in a group of Spanish schoolchildren*	
(3)	*Karimi-Hasanabad, 2014*	*Prevalence of vitamin d deficiency and its relationship with Body mass index and waist circumference in female Adolescents 17-14 years, boukan*	*Reported correlation coefficient for the relationship*
(4)	*Mellati, 2015*	*Vitamin D status and its associations with components of metabolic syndrome in healthy children*	
(5)	*Sioen, 2012*	*Determinants of vitamin D status in young children: results from the Belgian arm of the IDEFICS (Identification and Prevention of Dietary- and Lifestyle-Induced Health Effects in Children and Infants) Study*	
(6)	*Oliveira, 2014*	*Association of vitamin D insufficiency with adiposity and metabolic disorders in Brazilian adolescents*	*Reported mean±SD*
(7)	*Reis, 2009*	*Vitamin D status and cardiometabolic risk factors in the United States adolescent population*	*Duplicate report from population of another published study*
(8)	*Nam,2012*	*Estimate of a predictive cut-off value for serum 25-hydroxyvitamin D reflecting abdominal obesity in Korean adolescents*	

References:

1. *Al-Sadat N, Majid HA, Sim PY, Su TT, Dahlui M, Bakar MFA, et al. Vitamin D deficiency in Malaysian adolescents aged 13 years: findings from the Malaysian Health and Adolescents Longitudinal Research Team study (MyHeARTs). BMJ open. 2016;6(8):e010689.*
2. *Rodríguez-Rodríguez E, Navia-Lombán B, López-Sobaler A, Ortega R. Associations between abdominal fat and body mass index on vitamin D status in a group of Spanish schoolchildren. Eur J Clin Nutr. 2010;64(5):461-7.*
3. *Karimi-Hasanabad S, Rafraf M, Asghari-Jafarabadi M. Prevalence of vitamin d deficiency and its relationship with body mass index and waist circumference in female adolescents 17-14 years, Boukan. Iranian Journal of Diabetes and Metabolism. 2014;14(1):55-62.*
4. *Mellati AA, Sharifi F, Faghihzade S, Mousaviviri SA, Chiti H, Kazemi SAN. Vitamin D status and its associations with components of metabolic syndrome in healthy children. J Pediatr Endocrinol Metab. 2015;28(5-6):641-8.*
5. *Sioen I, Mouratidou T, Kaufman J-M, Bammann K, Michels N, Pigeot I, et al. Determinants of vitamin D status in young children: results from the Belgian arm of the IDEFICS (Identification and Prevention of Dietary-and Lifestyle-Induced Health Effects in Children and Infants) Study. Public Health Nutr. 2012;15(6):1093-9.*
6. *Oliveira RM, Novaes JF, Azeredo LM, Cândido APC, Leite IC. Association of vitamin D insufficiency with adiposity and metabolic disorders in Brazilian adolescents. Public Health Nutr. 2014;17(4):787-94.*
7. *Reis JP, Von Mühlen D, Miller ER, Michos ED, Appel LJ. Vitamin D status and cardiometabolic risk factors in the United States adolescent population. Pediatrics. 2009;124(3):e371-e9.*
8. *Nam GE, Kim DH, Cho KH, Park YG, Do Han K, Choi YS, et al. Estimate of a predictive cut-off value for serum 25-hydroxyvitamin D reflecting abdominal obesity in Korean adolescents. Nutr Res. 2012;32(6):395-402.*

Supplemental Table 3. *Details of quality assessment of included studies in the systematic review and met-analysis based on Newcastle-Ottawa Scale[1].*

	Representativeness of the sample	*Sample size*	*Non-respondents*	*Ascertainment of the exposure (risk factor)*	*Comparability of subjects in different outcome groups*	*Assessment of outcome*	*Statistical test*	*Total score*
Tang et al, 2020	*	*	*	**	**	*	*	*9*
Xiao et al, 2020	*	*	*	**	**	*	*	*9*
Fu et al, 2020	*	*	*	**	*	*	*	*8*
Kim et al, 2018	*	*		**	**	*	*	*8*
Cabral et al, 2016	*	*	*	**	*	*	*	*8*
Cediel et al, 2016	*	*	*	**	*	*	*	*8*
Al-Daghri et al, 2016	*	*	*	**		*	*	*7*
Al-Daghri et al, 2015	*	*	*	**	*	*	*	*8*
Jari et al, 2015	*	*	*	**	*	*	*	*8*
Belmonte et al, 2015	*		*	**		*	*	*6*
Lee et al, 2013	*		*	**	*	*	*	*7*
Lee et al, 2013	*	*	*	**		*	*	*7*
Nam et al, 2012	*	*	*	**	**	*	*	*9*
Pacifico et al, 2011			*	**	*	*	*	*6*

[1]Wells GA, Shea B, O'Connell D, Peterson J, Welch V, Tugwell P. The Newcastle-Ottawa Scale (NOS) for Assessing the Quality of Nonrandomised Studies in Meta-Analyses. Available from: http://www.ohri.ca/programs/clinical_epidemiology/oxford.asp
